# Systematic Identification of circRNA–miRNA–mRNA Regulatory Network in Esophageal Squamous Cell Carcinoma

**DOI:** 10.3389/fgene.2021.580390

**Published:** 2021-03-03

**Authors:** Yi Shen, Yi Shao, Chen Niu, Xiaoli Ruan, Zhaoping Zang, Rena Nakyeyune, Xiuhua Guo, Fen Liu

**Affiliations:** Department of Epidemiology and Health Statistics, School of Public Health, Beijing Municipal Key Laboratory of Clinical Epidemiology, Capital Medical University, Beijing, China

**Keywords:** esophageal squamous cell carcinoma, circular RNAs, ceRNA network, microarray, bioinformatics analysis

## Abstract

**Background:**

Circular RNAs (circRNAs) are described as endogenous non-coding RNAs that have been reported to play important roles in the development and progression of cancers. This study aimed to reveal the circRNA-related regulatory mechanism in esophageal squamous cell carcinoma (ESCC).

**Methods:**

A genome-wide circRNA microarray assay was performed to profile the expression of circRNAs in the blood of preoperative ESCC patients and healthy controls. A systematic method of data mining was performed to identify the differentially expressed miRNAs (DEmiRs) and differentially expressed genes (DEGs) based on the metaMA and RankProd analysis. Bioinformatics analyses and multiple tools were employed to construct the potential circRNA–miRNA–mRNA regulatory network.

**Results:**

Thirty-three differentially expressed circRNAs were identified in the ESCC blood, including 31 downregulated and two upregulated circRNAs in the blood of ESCC patients compared with the healthy controls. Twenty-three DEmiRs and 2,220 DEGs were obtained by the integration of microarray datasets. An ESCC-associated circRNA–miRNA–mRNA network was constructed based on 31 circRNAs, 3 DEmiRs, and 190 DEGs. Enrichment analyses indicated that the DEGs were associated with a series of biological processes and cancer-related pathways. The protein–protein interaction (PPI) network was generated by the 190 DEGs, with 10 hub genes verified in the network. Subsequently, a sub-network was established for ESCC, which included 29 circRNAs, 2 miRNAs, and 10 hub genes.

**Conclusion:**

Our study provided a novel clue to help understand the circRNA–miRNA–mRNA regulatory mechanism, highlighting the potential roles of circRNAs in the pathogenesis and development of ESCC.

## Introduction

Esophageal cancer is the seventh most common malignancy and the sixth leading cause of cancer-related deaths in the world (Bray et al., 2018). In China and most other Asian countries, esophageal squamous cell carcinoma (ESCC) is the predominant histologic subtype. The highest incidence of ESCC is found in certain areas surrounding the Taihang Mountains in North Central China (Chen et al., 2016). ESCC is frequently diagnosed at advanced stages and presents a poor prognosis. Despite the rapid advances in clinical diagnosis and treatment, the 5-year survival of patients with metastatic ESCC is still only 30.0% (Zeng et al., 2018). As a multifactorial disease, the complex genetic etiology and incomprehensive molecular pathogenesis may account for the clinical dilemma of ESCC. The specific molecular expression profiles will probably generate new clues for the diagnosis and treatment of cancers (Qu et al., 2017). Therefore, further exploration of novel molecular markers and their underlying mechanisms is extremely necessary to detect early ESCC and thus improve the prognosis for esophageal cancer patients.

Circular RNAs (circRNAs) are particular endogenous non-coding RNAs that are evolutionarily conserved across eukaryotic species and widely expressed in human cells with high abundance (Jeck et al., 2013). The closed loop structure makes circRNAs more stable and protects them from degradation by RNase R. With the increase in use of high-throughput sequencing and microarray technologies (Kristensen et al., 2018), more and more circRNAs have been shown to be associated with various diseases, especially cancer. Subsequent studies have discovered dysregulated circRNAs in the tissues from patients with different tumors (Vo et al., 2019). More importantly, they could function as microRNA (miRNA) sponges, protein decoys, and transporters to regulate gene expression (Qu et al., 2017). Current evidence has revealed that circRNAs are involved in tumor pathogenesis and progression by the competing endogenous RNA (ceRNA) regulatory network (Kristensen et al., 2018). Regarding ESCC, circRNA expression profiles have been investigated in cancerous tissues and esophageal cancer cells (Li et al., 2015a; Su et al., 2016; Xia et al., 2016; Sun et al., 2017; Wang et al., 2019). Some circRNAs have shown the ability to serve as diagnostic and prognostic biomarkers for ESCC (Niu et al., 2019). However, the functional roles and mechanisms of circRNAs in the development of ESCC remain unclear.

**FIGURE 1:**
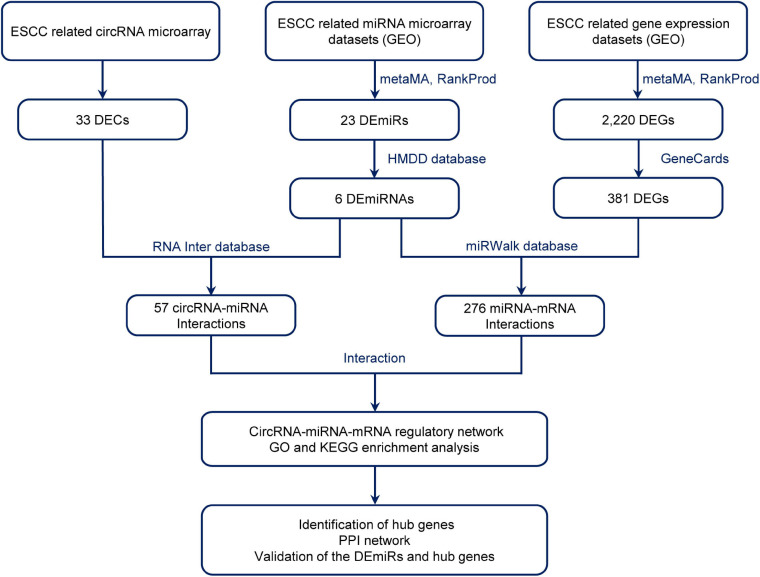
Flow diagram of the design and approaches of the present study. DECs, differentially expressed circular RNAs; DEmiRs, differentially expressed mircoRNAs; ESCC, esophageal squamous cell carcinoma; DEGs, differentially expressed genes; GEO, Gene Expression Omnibus; PPI, protein–protein interaction.

Therefore, the aim of this study was to explore the potential mechanisms of circRNA regulatory network in ESCC. We performed a genome-wide circRNA microarray assay and employed a comprehensive strategy to investigate differentially expressed circRNAs (DECs) and their potential mechanisms in ESCC. As the flow chart shows (Figure 1), to explore the sponge function of circRNAs in ESCC, we investigated the blood circRNAs expression profiles of patients with ESCC using circRNA microarray and collected the related circRNA–miRNA and miRNA–mRNA interactions by using the available databases, constructing a circRNA–miRNA–mRNA network. We then integrated ESCC-related microarray expression profiles from the Gene Expression Omnibus (GEO) database, obtaining differentially expressed miRNAs (DEmiRs) and genes (DEGs) by metaMA and RankProd method. Gene Oncology (GO) and Kyoto Encyclopedia of Genes and Genomes (KEGG) enrichment analyses for the target mRNAs were conducted to discover their potential functions. This provided a novel insight into the circRNAs and their functional mechanism in ESCC.

## Materials and Methods

### Study Subjects and Blood Sample Processing

Peripheral blood samples were collected from preoperative ESCC patients who underwent endoscopic submucosal dissection at the Cancer Hospital, Linzhou City, Henan Province in China, one of the high-risk areas of esophageal cancer, in January 2017. None of the patients received medical treatment before endoscopic examination or surgery. Each patient was diagnosed histopathologically. Frequency-matched healthy controls were selected from a cohort of attendees of the esophageal cancer early screening program during the same period (Table 1). This study was approved by the Ethics Committee of Capital Medical University (No. 2017SY22, Beijing, China). All recruited subjects provided written informed consent before participation. A double-blind approach was used throughout the entire research process. Five milliliters of venous blood (usually in the morning) was collected from each participant in an ethylenediaminetetraacetic acid (EDTA) tube. Blood samples were allowed to stand for 1 h at room temperature or kept at 4°C and processed within 24 h after collection. The blood samples were separated and then transferred into new tubes and stored at −80°C until use.

**TABLE 1:** Characteristics of ESCC patients and healthy controls.

Item ID	Sample Name	Group	Age (years)	Gender	RNA QC (Pass/Fail)
1	S1	ESCC patients	53	Male	Pass
2	S2	ESCC patients	54	Male	Pass
3	S4	ESCC patients	64	Female	Pass
4	S5	ESCC patients	61	Male	Pass
5	S6	ESCC patients	64	Female	Pass
6	S601057	Healthy controls	53	Male	Pass
7	S601193	Healthy controls	54	Male	Pass
8	S601049	Healthy controls	64	Female	Pass
9	S601050	Healthy controls	61	Female	Pass
10	S601137	Healthy controls	64	Male	Pass

*ESCC, esophageal squamous cell carcinoma; QC, quality control.*

### RNA Extraction

Total RNA was isolated from 1 ml of blood using TRIzol reagent (Invitrogen, Carlsbad, CA, United States) according to the manufacturer’s protocol. The integrity of RNA was evaluated by 1% denaturing agarose gel electrophoresis. The concentration and purity of total RNA was then measured with the NanoDrop ND-1000 spectrophotometer (Thermo Fisher Scientific, Wilmington, DE, United States).

### CircRNA Microarray

Total RNA from each sample was digested using Ribonuclease R (RNase R; Epicenter, Madison, WI, United States) to remove linear RNAs and to enrich circular RNAs. The Super RNA Labeling Kit (Arraystar, Rockville, MD, United States) was then used to amplify and transcribe the circRNAs into fluorescently labeled cRNAs by using random primers. The fluorescently labeled cRNAs were then purified with an RNeasy Mini Kit (Qiagen, Hilden, Germany). The concentrations of fluorescently labeled cRNAs (pmol Cy3/μg cRNA) were then measured with a NanoDrop ND-1000 spectrophotometer (Thermo Fisher Scientific, Wilmington, DE, United States). Based on calculated concentrations, 1 μg of fluorescently labeled cRNA for each sample was fragmented by incubation with 10 × blocking agent (5 μl) and 25 × fragmentation buffer (1 μl) at 60°C for 30 min. Next, 2 × hybridization buffer (25 μl) was added to the reaction. The resulting hybridization solution (50 μl) was placed in a gasket slide, which was then assembled with the Arraystar Human CircRNA Microarray Slides V2.0 (8 × 15K; Arraystar), with a total of 13,617 circRNA probes on the microarray, according to the kit’s instructions. The slides were then incubated in an Agilent Hybridization Oven at 65°C for 17 h. The hybridized microarrays were then washed, fixed, and scanned with an Agilent G2505C Scanner (Agilent Technologies, Santa Clara, United States).

### Microarray Data Analysis

The acquired array images were imported into Agilent Feature Extraction software (version 11.0.1.1; Agilent Technologies) for raw data extraction. Quantile normalization of raw data and subsequent data processing were performed by using the “limma” package of R software (R software package, version 3.1.2)^1^. DECs with statistical significance [fold change (FC) > 2 and *P*-value < 0.05] between two groups were identified through fold change filtering or Volcano plot filtering. Hierarchical clustering was performed to show the distinguishable circRNA expression pattern among samples. The microarray raw data in this study were deposited in the GEO database^2^ and are accessible by the GEO accession number GSE112496.

### DEmiRs and DEGs in ESCC

Microarray datasets of miRNA and gene expression profiles of ESCC were obtained from the GEO database. Eligible datasets had to meet the inclusion criteria: (1) microarray profile studies on patients with ESCC; (2) using cancerous tissues and normal tissues for comparison; (3) indicating the classification of each biological sample (tumor or normal); (4) including annotated information (gene symbol or miRNA ID) for each probe in the microarray. We obtained four miRNA expression profiles (GSE59973, GSE97049, GSE114110, and GSE145198) and four gene expression profiles (GSE29001, GSE20347, GSE38129, and GSE23400) of ESCC from GEO database, respectively. All the expression data were normalized and log2 transformed. Next, all microarray probe IDs were converted to miRNA IDs/or Entrez Gene IDs. When multiple probes had identical miRNA IDs/or Entrez Gene IDs, we selected the probe that presented the mean interquartile range to represent the miRNA IDs/or Entrez Gene IDs. We integrated the different microarray datasets using the R-based “metaMA” package (Marot et al., 2009) and “RankProd” package (Carratore et al., 2017). “metaMA” package can be applied to multiple microarray datasets to extract differentially expressed genes. MetaMA provided a method to calculate combined *P*-values obtained from limma procedures (Marot et al., 2009), while RankProd identified the differentially expressed genes by a non-parametric rank product method (Carratore et al., 2017). The overlapping miRNAs and genes from the metaMA (combined *P*-value < 0.05) and RankProd (*P*-value < 0.05, FC > 1.5) methods were identified as DEmiRs and DEGs.

Furthermore, we manually searched the Human MicroRNA Disease Database (HMDD) and GeneCards database. HMDD database is a comprehensive database that provides evidence of miRNA–disease associations supported by published literature. It is freely accessible at http://www.cuilab.cn/hmdd (Lu et al., 2008). GeneCards integrates comprehensive information on all annotated and predicted human genes. A search engine at the top of the GeneCards homepage provides access to detailed information about genes using a keyword query (Stelzer et al., 2016). The associations between ESCC and miRNAs based on literature-derived evidence code were downloaded directly from the HMDD database. Also, the ESCC-associated genes were retrieved from the results of the key word “esophageal squamous cell carcinoma” OR “ESCC” query term. We then determined the ESCC-related DEmiRs and DEGs by intersecting the identified DEmiRs and DEGs with the known ESCC-associated miRNAs and genes in HMDD and GeneCards databases.

### Prediction of miRNAs for circRNAs and Target Genes for DEmiRs

The circRNA–miRNA interactions were predicted by using the RNA Interactome Database (RNAInter)^3^, which provides accurate prediction of RNA–RNA interactions by the input sequence of DECs and DEmiRs (Lin et al., 2020). RNAInter is a comprehensive RNA interaction database that recruits experimental and computational prediction RNA-associated interactions from literature and 35 other resources, including more than 41 million RNA–RNA interactions in 154 species. We filtered the circRNA–miRNA interactions with more than 7 base pairs in the seed. The target genes of miRNAs were derived by using miRWalk 3.0, an integrated database that provides miRNA–target gene interactions (Sticht et al., 2018). We predicted the target genes that were combined in the seed area and selected the miRNA–target gene interactions with a cutoff score of 0.95.

### Construction of circRNA–miRNA–mRNA Network in ESCC and Functional Analysis

To further investigate the potential regulatory mechanism, we extracted the circRNA–miRNA–mRNA competitive binding relationships. The circRNA–miRNA and miRNA–mRNA interactions obtained from the databases were intersected with the ESCC-related DEmiRs and DEGs, and the overlapping DEmiRs and DEGs were retained for further network analysis. The selected DECs, DEmiRs, and DEGs were then mapped to construct a circRNA–miRNA–mRNA interaction network, which was visualized using Cytoscape 3.8.2 software^4^.

### Functional Enrichment Analysis

For further insight into the putative gene functions and pathways of the target genes, we performed the GO and KEGG pathway enrichment analyses using Metascape Bioinformatics Resources^5^ (Zhou et al., 2019). All the significant GO terms and KEGG pathways were identified with the adjusted *P*-values (adj. *P*-values) < 0.05, which were corrected by the Benjamini–Hochberg method. We then calculated the −log_10_ (adj. *P*-values) for the significant GO terms and KEGG pathways. The higher the −log_10_ (adj. *P*-values) were, the more significant the terms.

### Identification of Hub Genes in the Protein–Protein Interaction (PPI) Network

After the intersection of the circRNA–miRNA, miRNA–mRNA interactions and the ESCC-related DEmiRs and DEGs, we obtained a series of overlapping DEmiRs and DEGs. To evaluate the protein–protein interaction (PPI) relationships among overlapping DEGs, we calculated the interaction scores of the DEGs and constructed a PPI network by using Search Tool for the Retrieval of Interacting Genes/Proteins (STRING)^6^. The DEGs with an interaction score >0.4 were included in the PPI network. In addition, Maximal Clique Centrality (MCC) and degree algorithms were applied to identify the hub genes in the PPI network. We selected the top 10 DEGs with highest MCC and degree scores as hub genes in the network.

### Validation of the DEmiRs and DEGs in the Hub Network

MiRCancer^7^ is a miRNA cancer association database that comprehensively collects miRNA expression profiles in human cancers from published literature. The database of Differentially Expressed MiRNAs in human Cancers (dbDEMC 2.0) is an integrated database that stores and displays aberrant miRNAs in human cancers detected by high-throughput methods^8^. We searched the DEmiRs in miRCancer and dbDEMC databases to validate their differential expression.

The gene expression data were downloaded from The Cancer Genome Atlas (TCGA) database using UCSC Xena^9^, which is an online database for analyzing gene expression profiles from the TCGA and the genotype-tissue expression (GTEx) projects. We validated the expression levels of hub genes in esophageal cancer (EC) tissues and esophageal normal tissues using the gene expression data from TCGA and GTEx databases. Furthermore, we obtained the survival map of the hub genes from GEPIA^10^, which is an online database that contains gene expression profiles from the TCGA and GTEx databases as well as provides survival analyses for gene expression profiles in cancers. The Cox proportional hazard ratios (HRs) were calculated to compare the risk of death between patients with high gene expression and low gene expression group (as reference), which were divided by medians.

## Results

### Overview of circRNA Expression Profiles

**FIGURE 2:**
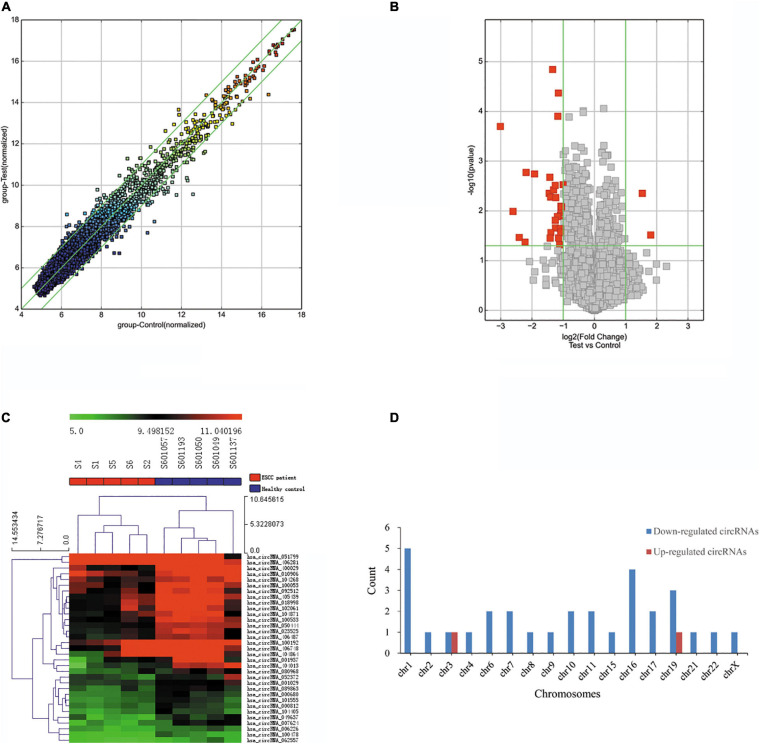
Differentially expressed circRNAs in ESCC patients. **(A)** The scatter plot of differentially expressed circRNAs. Green lines represent fold change of 2.0. **(B)** Volcano plot of differentially expressed circRNAs. The green vertical lines correspond to a fold change of 2.0, and the green horizontal line corresponds to a *P*-value of 0.05. **(C)** Hierarchical clustering of differentially expressed circRNAs. Each column represents a sample and each row represents a circRNA. The color reflects the expression level of circRNAs and changes from red (high) to black (medium) to green (low). **(D)** Bar plot shows the distributions of the differentially expressed circRNAs in human chromosomes.

The blood circRNA expression profiles of ESCC patients and healthy controls were quantified by a high-throughput human circRNA microarray platform that comprises 13,617 probes. In total, 33 significant differentially expressed circRNAs were identified (FC > 2.0 and *P*-value < 0.05), among which two (6.06%) were upregulated and the other 31 (93.94%) were downregulated in ESCC patients (Table 2), which suggested that downregulated circRNAs were more prevalent than upregulated ones in the microarray data. A scatter plot (Figure 2A) and a volcano plot (Figure 2B) of all detectable circRNAs were used to better demonstrate the significantly and differentially expressed circRNAs. Hierarchical clustering showed that expression levels of DECs were consistent within each group but evidently different between the ESCC patients and control groups (Figure 2C). Moreover, most of the downregulated circRNAs were transcribed from the protein encoding sequences located in chr1, chr16, and chr19, while the two upregulated ones were located in chr3 and chr19, respectively (Figure 2D).

**TABLE 2:** Differentially expressed circRNAs in the plasma of ESCC patients.

circRNAs	*P*-value*	FC	Deregulation	Chromosome	GeneSymbol
hsa_circRNA_406281	0.0044	2.8947389	Up	chr3	GNL3
hsa_circRNA_051799	0.0305	3.487203	Up	chr19	BAX
hsa_circRNA_400029	0.0002	8.07711	Down	chr19	RPL13
hsa_circRNA_404013	0.0102	6.1026306	Down	chr16	BRF2
hsa_circRNA_001937	0.0340	5.3070016	Down	chr22	CHD9
hsa_circRNA_404864	0.0423	4.6631225	Down	chr6	GAS2
hsa_circRNA_007624	0.0017	4.5571772	Down	chr1	BCAR3
hsa_circRNA_049637	0.0018	3.7794029	Down	chrX	CALR
hsa_circRNA_100053	0.00448	2.7324244	Down	chr19	MFN2
hsa_circRNA_405439	0.00218	2.6883651	Down	chr11	SNX29
hsa_circRNA_080968	0.035288	2.6710479	Down	chr1	ADAM22
hsa_circRNA_089863	0.0053	2.6485388	Down	chr4	SHROOM2
hsa_circRNA_406748	0.0272	2.6311882	Down	chr19	ZFP57
hsa_circRNA_050444	0.00001	2.5258589	Down	chr21	GPATCH1
hsa_circRNA_023525	0.0038	2.4734212	Down	chr7	UCP2
hsa_circRNA_000680	0.0031	2.3872937	Down	chr2	IQCK
hsa_circRNA_018998	0.0155	2.3833125	Down	chr10	CCSER2
hsa_circRNA_104405	0.0220	2.377988	Down	chr15	GATSL1
hsa_circRNA_406487	0.0054	2.3610631	Down	chr8	UBA6-AS1
hsa_circRNA_101555	0.0130	2.2697815	Down	chr1	CSNK1G1
hsa_circRNA_100533	0.0001	2.2577997	Down	chr1	PFKP
hsa_circRNA_001029	0.00004	2.2216968	Down	chr16	DYSF
hsa_circRNA_010906	0.0348	2.2184354	Down	chr6	IFNLR1
hsa_circRNA_052372	0.0481	2.1664142	Down	chr11	TRIM28
hsa_circRNA_102061	0.0226	2.1279783	Down	chr1	MED1
hsa_circRNA_104268	0.0124	2.1267317	Down	chr16	WDR27
hsa_circRNA_104871	0.0267	2.1151064	Down	chr3	SUSD1
hsa_circRNA_100478	0.0094	2.1068894	Down	chr16	C1orf198
hsa_circRNA_062557	0.0080	2.1038778	Down	chr7	CHCHD10
hsa_circRNA_000812	0.0082	2.0753054	Down	chr10	RNF213
hsa_circRNA_092512	0.0107	2.0735833	Down	chr17	ITGB2
hsa_circRNA_100192	0.0258	2.0547516	Down	chr17	ST3GAL3
hsa_circRNA_006226	0.0030	2.0067748	Down	chr9	PTPRG

circRNA, circular RNA; ESCC, esophageal squamous cell carcinoma; FC, fold change. *P-value, *P*-value is calculated from student *t*-test. circRNAs with FC > 2.0 and *P*-value < 0.05 are identified to be differentially expressed.

### Identification of DEmiRs and DEGs in ESCC

Four miRNA microarray datasets (GSE59973, GSE97049, GSE114110, and GSE145198) and four gene expression profiles (GSE29001, GSE20347, GSE38129, and GSE23400) were, respectively, integrated in our study. All these expression profiles were investigated in the samples of ESCC tissues and normal tissues from Chinese populations. Also, several of the datasets (GSE145198, GSE29001, GSE20347, GSE38129, and GSE23400) were obtained from populations in high ESCC incidence regions of China. The microarray assays were generated from the Agilent platform and Affymetrix platform. Detailed information of the four datasets is presented in Supplementary Table 1. Normalization of raw expression data was performed before further analyses. Boxplots of normalized expression data for all datasets are shown in Supplementary Figure 1. A total of 23 DEmiRs were determined by metaMA (combined *P*-value < 0.05) and RankProd (*P*-value < 0.05, FC > 1.5) methods, including 10 upregulated and 13 downregulated DEmiRs. The detailed information of DEmiRs is shown in Supplementary Table 2 and Supplementary Figure 2.

As for the gene expression profiles (GSE29001, GSE20347, GSE38129, and GSE23400), 2,220 DEGs were identified through the metaMA and RankProd method, including 1,209 upregulated and 1,011 downregulated DEmiRs. The detailed information of DEGs is shown in the Supplementary Table 3 and Supplementary Figure 2. The identified DEmiRs and DEGs were further selected from HMDD and GeneCards databases. After the intersection with the known ESCC-associated miRNAs and genes in the databases, six ESCC-related DEmiRs (three upregulated DEmiRs and three downregulated DEmiRs) and 381 DEGs (104 upregulated DEGs and 277 downregulated DEGs) were determined.

### Identification of circRNA–miRNA and miRNA–mRNA Interactions

We collected the interacted circRNAs and mRNAs for the six ESCC-related DEmiRs from two online databases, RNA Inter and miRWalk. There were 57 circRNA–miRNA interactions in the RAID database and 423 miRNA–mRNA interactions in the miRWalk database. These miRNA–mRNA interactions were intersected with the 381 ESCC-related DEGs, and 190 overlapping DEGs were extracted for further network analysis in our study.

### Construction of circRNA–miRNA–mRNA Network

**FIGURE 3:**
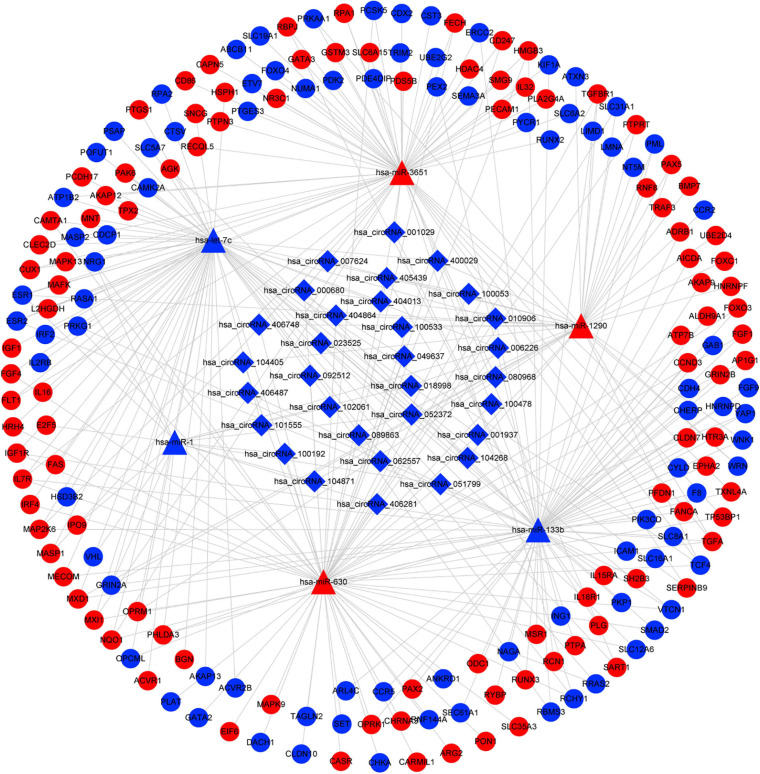
CircRNA–miRNA–mRNA interaction network in ESCC. CircRNAs are represented by diamonds; miRNAs are shown as triangles; the DEGs are represented by ellipses. Red represents upregulated expression, and blue color represents downregulated expression. circRNA, circular RNA; miRNA, microRNA; ESCC, esophageal squamous cell carcinoma; DEGs, differentially expressed genes.

Based on the identified circRNA–miRNA and miRNA–mRNA interactions, we constructed a circRNA–miRNA–mRNA network to visualize the interactions between the selected circRNAs, the ESCC-related DEmiRs, and DEGs. The miRNA-connected regulatory relationship depended on the number of shared DEmiRs. As shown in Figure 3, this regulatory network included 31 DECs, 6 DEmiRs and 190 DEGs. Hsa-let-7c and hsa-miR-133b exhibited the highest number of circRNA and mRNA binding sites, indicating that they may be competitively sponged by circRNAs and thus exert essential roles in the regulatory network. The details of the interaction network are listed in Supplementary Table 4.

### Functional Analysis

**FIGURE 4:**
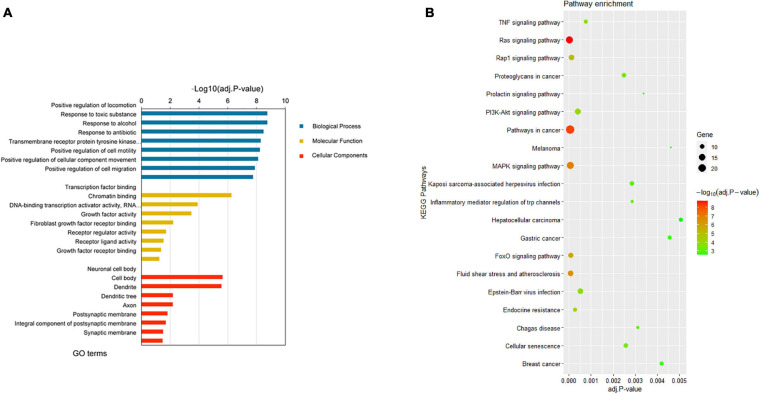
Enrichment analyses results of the DEGs from the network. **(A)** Top 8 enriched GO terms (biological process, cellular component, and molecular function) of DEGs. **(B)** Top 20 enriched KEGG pathway of DEGs. DEGs, differentially expressed genes; GO, gene ontology; KEGG, Kyoto Encyclopedia of Genes and Genomes.

Gene Oncology and KEGG pathway enrichment analyses were then performed to further explore the potential functions of the 190 target DEGs involved in the network. According to the GO enrichment results, the overlapping target genes were significantly enriched in biological processes including positive regulation of locomotion, positive regulation of cell motility, positive regulation of cell migration, positive regulation of cellular component movement, and so on (Figure 4A). Results of KEGG pathway enrichment analysis revealed that the target genes were enriched in several cancer-related signaling pathways, namely, Ras signaling pathway, MAPK signaling pathway, FoxO signaling pathway, PI3K-Akt signaling pathway, TNF signaling pathway, gastric cancer, melanoma, and hepatocellular carcinoma (Figure 4B). More information on the enrichment analyses is presented in Supplementary Table 5.

### Validation of the DEmiRs and Hub Genes in the Hub Network

According to the search results from miRCancer and dbDEMC databases, three DEmiRs (hsa-miR-1, hsa-let-7c, and hsa-miR-133b) were significantly downregulated in EC, while two DEmiRs showed significant overexpression in EC (Table 3), which was consistent with the results of the metaMA and RankProd analyses from the GEO datasets.

**TABLE 3:** Validation of DEmiRs in the public databases.

DEmiRs	meta MA + RankProd	dbDEMC 2.0	miRcancer
hsa-miR-1	Down	Down	Down
hsa-let-7c	Down	Down	Down
hsa-miR-133b	Down	Down	Down
hsa-miR-1290	Up	Up	Up
hsa-miR-3651	Up	NA	NA
hsa-miR-630	Up	Up	Down

*DEmiRs, differentially expressed miRNAs; NA, not available.*

**FIGURE 5:**
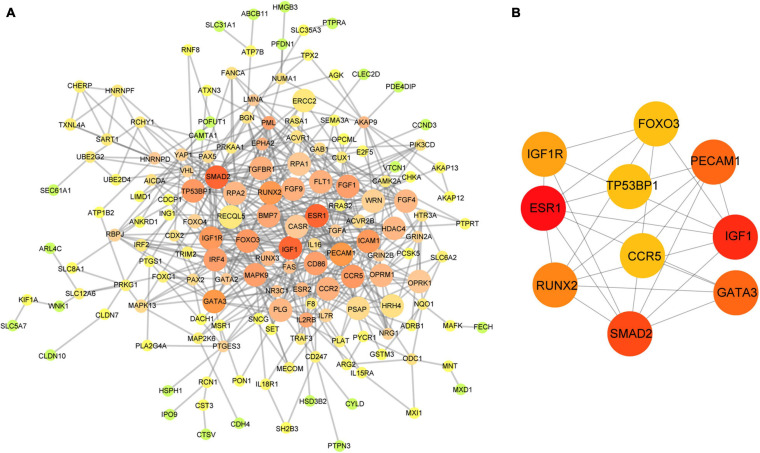
Identification of hub genes from the PPI network. **(A)** The PPI network of 190 DEGs in the network; the edge size and color change gradually in ascending order according to the degree of the genes. **(B)** The 10 hub genes identified by MCC and degree scores. PPI, protein–protein interaction; DEGs, differentially expressed genes; MCC, Maximal Clique Centrality.

**FIGURE 6:**
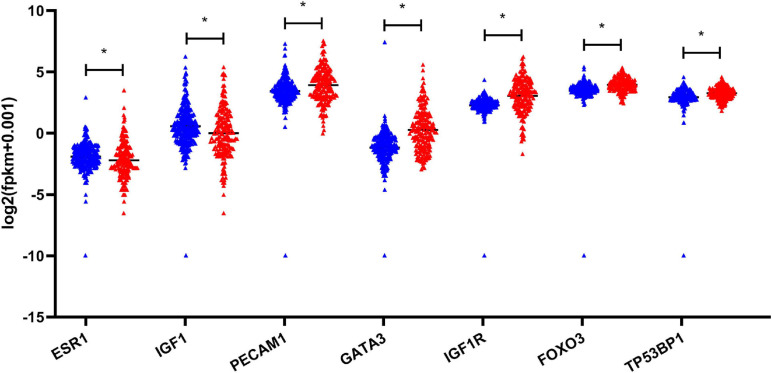
Validation of the expression of hub genes based on TCGA and GTEx database. *P-value < 0.05. EC, esophageal carcinoma; TCGA, The Cancer Genome Atlas; GTEx, the genotype-tissue expression projects.

**FIGURE 7:**
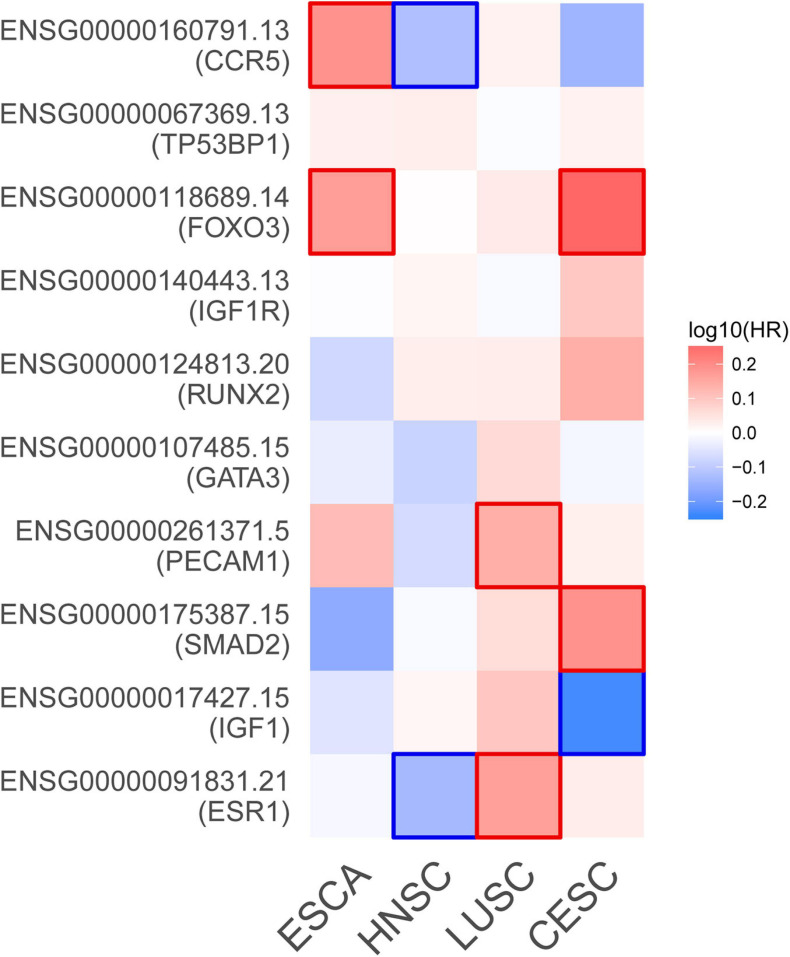
Survival map of the hazard ratios. The significant results (*P*-value < 0.05) of hub genes are marked in the map. CESC, cervical squamous cell carcinoma and endocervical adenocarcinoma; ESCA, esophageal carcinoma; HNSC, head and neck squamous cell carcinoma; LUSC, lung squamous cell carcinoma.

**FIGURE 8:**
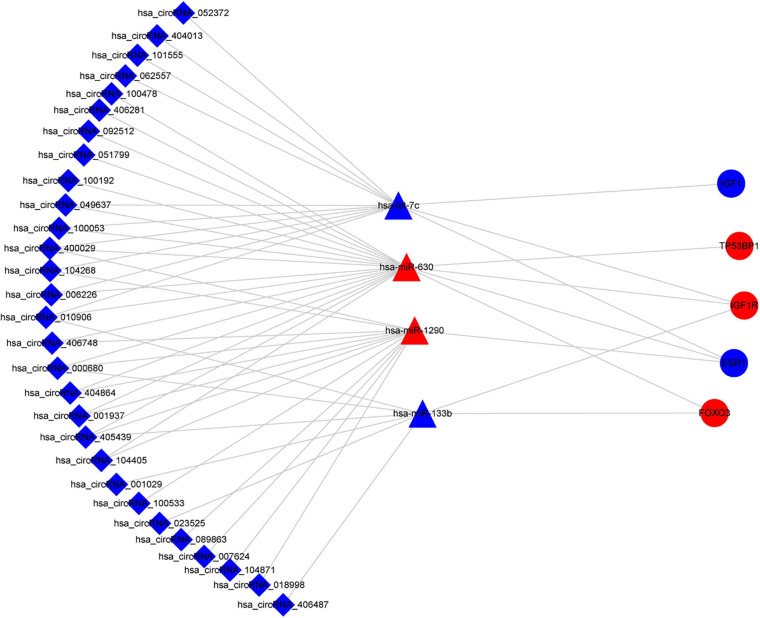
Sub-network of circRNAs, miRNAs, and hub genes. circRNAs are represented by diamonds; miRNAs are shown as triangles; hub genes are represented by ellipses. Red represents upregulated expression, and blue color represents downregulated expression. circRNA, circular RNA; miRNA, microRNA.

Based on the aforementioned investigation, we established a PPI network consisting of 160 nodes and 296 edges to excavate the interrelationship among the 190 DEGs (Figure 5A). A module consists of 10 hub genes with the highest MCC and degree scores, which is shown in Figure 5B. We validated the expression levels of the 10 hub genes using gene expression data from TCGA and GTEx databases. Two of the hub genes (ESR1 and IGF1) were confirmed to be under-expressed in EC tissues, and five of the hub genes (PECAM1, GATA3, IGF1R, FOXO3, and TP53BP1) were validated to be over-expressed in EC tissues (Figure 6). Furthermore, a survival plot showed that the high expression of some hub genes (FOXO3, CCR5, PECAM1, ESR1, and SMAD2) was associated with high risk of death in patients with esophageal carcinoma and other squamous cell carcinomas (Figure 7). A sub-network was then constructed to delineate the interactions among DECs, validated DEmiRs, and validated hub genes (Figure 8). There were four regulatory modules found according to the sub-network, including 29 DECs, 4 DEmiRs, and 5 DEGs.

## Discussion

As a special class of covalently closed RNA molecules, circRNAs have been revealed more stable than linear RNAs and have multiple functions in the pathogenesis of different conditions. It has been well accepted that circRNAs could act as the endogenous miRNA “sponge” to competitively absorb miRNAs and thus play important roles in post-transcriptional regulation. Recently, research has focused on the dysregulated circRNAs and their functions in the development of esophageal cancer (Li et al., 2015a; Xia et al., 2016; Fan et al., 2019). Several circRNAs were involved in multiple biological processes of ESCC, such as cell proliferation, proliferation, migration, and invasion (Rong et al., 2018; Sang et al., 2018; Hu et al., 2019; Wang et al., 2019; Xing et al., 2020). However, the functional mechanism of circRNAs in ESCC still remains largely unknown and needs to be investigated. In this study, we first identified a comprehensively dysregulated expression profile of blood circRNAs in ESCC. A total of 33 aberrantly expressed circRNAs (FC > 2.0 and *P*-value < 0.05) were identified using a microarray-based assay, including 2 upregulated and 31 downregulated circRNAs (Table 2). Moreover, we identified the circRNA-associated ceRNA network in ESCC through a comprehensively integrated strategy. Microarray expression profiles in different platforms were collected from the GEO database. In addition, metaMA and RankProd analyses were performed to identify the DEmiRs and DEGs in ESCC tissues and normal tissues. Based on the circRNA microarray profiles and the public microarray datasets, the circRNA–miRNA–mRNA regulatory network was constructed, which could provide clues for the ceRNA mechanism in ESCC. Furthermore, we explored the functions and pathways of DEGs in the network through the application of bioinformatics analysis tools.

In our study, most of the differentially expressed circRNAs are transcribed from the human protein-coding exons. Hsa_circRNA_007624 was the most downregulated circRNA and transcribed from the *BCAR3* gene, which encodes proteins to induce the resistance to antiestrogens of breast cancer cells (van Agthoven et al., 1998). We speculated that hsa_circRNA_007624 might be involved in the initiation and development of cancer. We also identified two DEmiRs (hsa-let-7c and hsa-miR-1290) that were associated with ESCC, which exhibited consistency with previous studies. Downregulation of hsa-let-7c has been shown to be correlated with poor prognosis of ESCC patients (Sugimura et al., 2012). The aberrant expression of hsa-miR-1290 has been previously reported in ESCC tissue samples, which was associated with the cell proliferation and metastasis in ESCC (Li et al., 2015b; Mao et al., 2015). However, further validation is necessary to detect the expression pattern of these circRNAs and miRNAs in ESCC.

Based on the integrated expression data and target gene predication, we next explored the potential functions and pathways of the 190 ESCC-related DEGs. GO analysis suggested that the target genes were involved in a series of processes, including regulation of cell motility, cell migration, and cellular component movement (Figure 4A). For the results of KEGG enrichment analysis, these ESCC-related DEGs were significantly involved in gastric cancer, melanoma, and hepatocellular carcinoma pathways (Figure 4B). In addition, the FoxO signaling pathway regulates a broad range of cellular functions that are crucial to tumorigenesis, including cell proliferation and apoptosis (Myatt and Lam, 2007). It has been reported that the MAPK signaling pathway plays an important role in cancer cell proliferation (Qiu et al., 2020) and tumor metastasis (Cheng et al., 2020). The PI3K-Akt signaling pathway is known as an oncogenic pathway and is associated with tumor angiogenesis, growth, and survival (Noorolyai et al., 2019).

To further clarify the regulatory mechanism of the circRNA–miRNA–mRNA network, we identified the 10 hub genes from the PPI network of 190 DEGs and then constructed the circRNA–miRNA–hub gene interaction network (Figure 5). Several of the hub genes (GATA3, IGF1R, and FOXO3) have been reported to be associated with the tumor progression of ESCC in previous studies. GATA3 was observed to directly regulate the transcriptional repression of androgen receptor, which exerted oncogenic functions in ESCC (Huang et al., 2020). FOXO3 was known as an inhibitor of cancer-related cell cycle progression and also contributed to the proliferation and metastasis of ESCC cells (Lu et al., 2018). IGF1R was significantly associated with the inhibition of the proliferation, migration, and invasion in ESCC cells (Mei et al., 2017). In addition, CCR5 showed a high expression pattern in ESCC tissues and was associated with poor prognosis (Kodama et al., 2020). Moreover, our enrichment analysis revealed that the aforementioned genes were all enriched in the cancer-related signaling pathway. Therefore, according to the sub-network, we suspected that some circRNA–miRNA–mRNA axes, such as hsa_circRNA_007624–hsa-miR-1290–ESR1, hsa_circRNA_100053–has-let-7c–ESR1/IGF1/IGF1R, and hsa_circRNA_100053–hsa-miR-630–ESR1/TP53BP1/IGF1R/FOXO3, indicated a competitive interaction network of circRNAs in ESCC. The aforementioned evidence might provide insights into the regulatory mechanism in the development of ESCC at the molecular level. However, these findings are based on microarray profiles and public databases. Additional experiments, such as qRT-PCR, luciferase reporter assay, and the main effects of key circRNAs on biological processes are indispensable to further validate the possible roles of the regulatory network in ESCC.

There is an increasing amount of available microarray research on ESCC, although most datasets were performed independently and with relatively small samples. One of the strengths of our study is that it systematically integrated different microarray data to better identify the differentially expressed miRNAs and genes utilizing metaMA and RankProd analyses. This increased the sensitivity to reveal the deregulated miRNA and genes in ESCC. However, several limitations still needed to be clarified in this study. First, this study was based on a comprehensive strategy of differential expression profiles but did not confirm the expression levels of the circRNAs, miRNAs, and mRNAs in the same samples, thus validation in large cohorts is necessary to verify their co-expression patterns in ESCC. Second, although we have predicted the regulatory functions and pathways for the ceRNA network based on the multiple reliable algorithms, more vivo or vitro studies are required to investigate the oncogenic or tumor-suppressed functions of circRNAs in the development of ESCC.

## Conclusion

Our study constructed a potential circRNA–miRNA–mRNA network in ESCC based on the integration of microarray datasets and bioinformatics analysis. This regulatory network suggests that circRNAs may be involved in the cancer-related signaling pathways through functioning as miRNA sponges. These findings will serve as a basis for the diagnostic and therapeutic application of circRNAs in ESCC. Further research is needed to evaluate the potential diagnostic value of circulating circRNAs in ESCC and further reveal its underlying mechanism.

## Data Availability Statement

The data included in our study can be found in the Gene Expression Omnibus (GEO) repository by the GEO accession number GSE112496.

## Ethics Statement

The studies involving human participants were reviewed and approved by the Ethics Committee of Capital Medical University (No. 2017SY22, Beijing, China). The patients/participants provided their written informed consent to participate in this study.

## Author Contributions

FL conceived and designed the study, reviewed and revised the article, and made the decision to submit for publication. YShe, XR, ZZ, and RN collected the data. YShe, YSha, and CN analyzed the data. YShe, FL, and XG drafted the article. All authors have read the article and approved the submitted version.

## Conflict of Interest

The authors declare that the research was conducted in the absence of any commercial or financial relationships that could be construed as a potential conflict of interest.

## Footnotes

1 http://www.r-project.org/

2 https://www.ncbi.nlm.nih.gov/geo/info/linking.html

3 http://www.rna-society.org/rnainter/

4 http://www.cytoscape.org/

5 https://metascape.org/

6 https://string-db.org/

7 http://mircancer.ecu.edu/

8 https://www.picb.ac.cn/dbDEMC/index.html

9 http://xena.ucsc.edu/

10 http://gepia.cancer-pku.cn/

## Supplementary Material

Supplementary Figure 1 Box plots for miRNA and gene expression datasets after normalization.

Supplementary Figure 2 Venn diagrams of DEmiRs and DEGs from metaMA analysis. Different colors represent the different datasets. The crossed areas indicate the overlapping molecules. The circles in the Venn diagram represent each individual dataset and combined datasets for metaMA analysis. The values in the dataset circles show the numbers of DEmiRs and DEGs identified from each individual dataset and combined metaMA analysis. **(A)** Identified DEmiRs from the GEO datasets: GSE59973, GSE97049, GSE114110, and GSE145198. **(B)** Identified DEGs from the GEO datasets: GSE29001, GSE20347, GSE38129, and GSE23400. DEmiRs, differentially expressed microRNAs; DEGs, differentially expressed genes; GEO, Gene Expression Omnibus.

Supplementary Table 1 Detailed information of the microarray datasets from GEO database.

Supplementary Table 2 Differentially expressed miRNAs identified by metaMA and RankProd analysis.

Supplementary Table 3 Differentially expressed genes identified by metaMA and RankProd analysis.

Supplementary Table 4 Interactions of circRNA–miRNA–mRNA network.

Supplementary Table 5 Enrichment analyses results of the DEGs from the network.
