# Supplementary material for: Systematic Identification of circRNA–miRNA–mRNA Regulatory Network in Esophageal Squamous Cell Carcinoma
Source: Front Genet. 2021 Mar 3;12:580390. doi: 10.3389/fgene.2021.580390 (PMC7966720; doi:10.3389/fgene.2021.580390)
Supplement: Supplementary Figure 1 — Box plots for miRNA and gene expression datasets after normalization. [file Data_Sheet_1.zip › Supplementary Table 1.docx]

| Table S1 Detailed information of the microarray datasets from GEO database | | | | | | |
| --- | --- | --- | --- | --- | --- | --- |
| Accession ID | Platform | Contributor(s) | Year | Sample size (T/N) | Area | Microarray type |
| GSE59973 | GPL16770 | Shi R | 2015 | 3 / 3 | China | miRNA |
| GSE97049 | GPL21572 | Lin C, et al. | 2017 | 7 / 7 | China | miRNA |
| GSE114110 | GPL24967 | Wen J, et al. | 2018 | 30 / 10 | China | miRNA |
| GSE145198 | GPL18044 | Liu F, et al. | 2020 | 4 / 4 | China | miRNA |
| GSE29001 | GPL571 | Yan W, et al. | 2011 | 36 / 9 | China | Gene |
| GSE20347 | GPL571 | Clifford RJ, et al. | 2011 | 17 / 17 | China | Gene |
| GSE38129 | GPL571 | Hu N, et al. | 2015 | 30 / 30 | China | Gene |
| GSE23400 | GPL96 | Su H, et al. | 2010 | 53 / 53 | China | Gene |

GEO: Gene Expression Omnibus; T: tumor tissue of esophageal squamous cell carcinoma; N: normal tissue; miRNA: microRNA
