# Supplementary figures and images for: Systematic Identification of circRNA–miRNA–mRNA Regulatory Network in Esophageal Squamous Cell Carcinoma
Source: Front Genet. 2021 Mar 3;12:580390. doi: 10.3389/fgene.2021.580390 (PMC7966720; doi:10.3389/fgene.2021.580390)

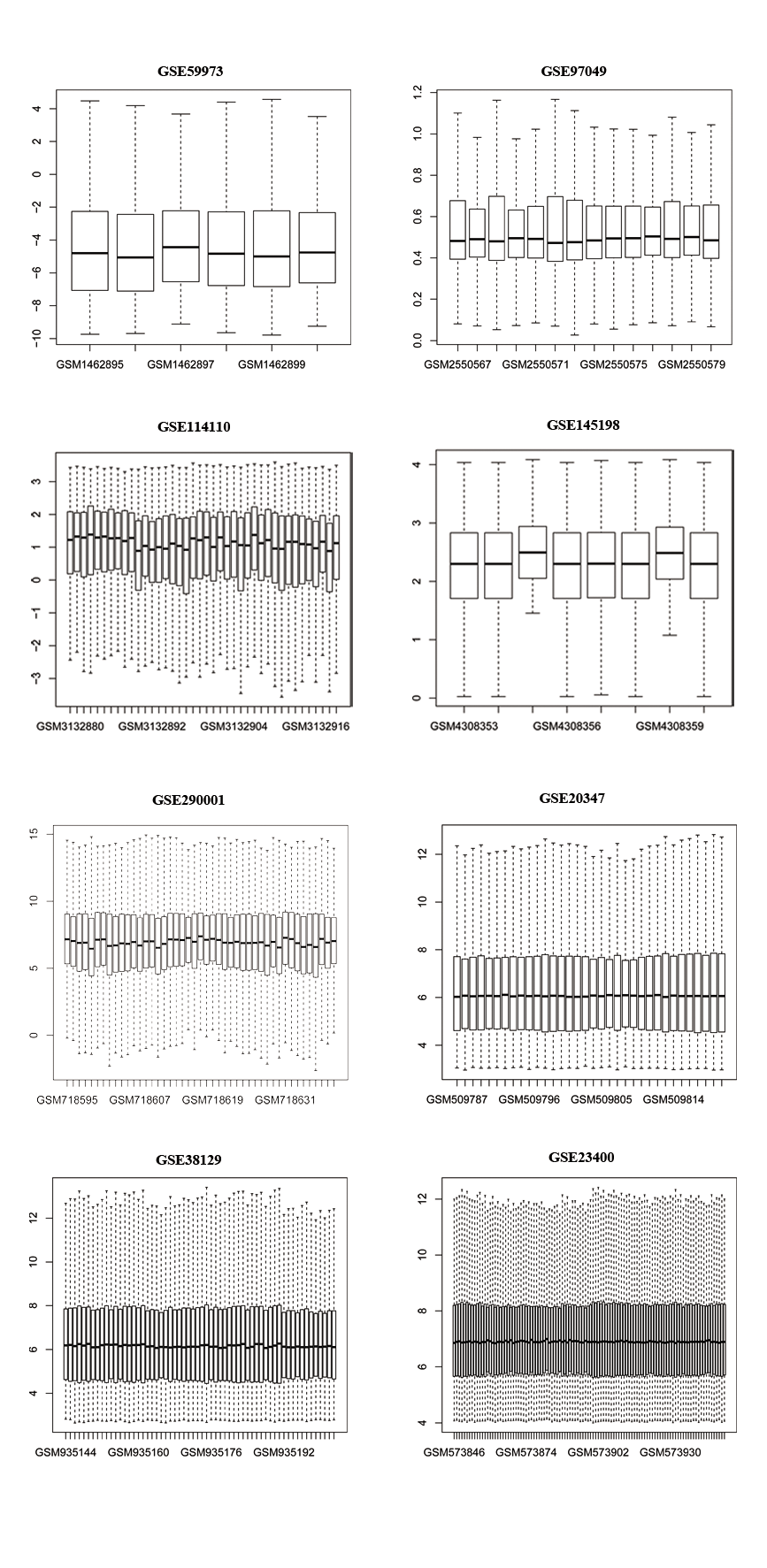

Supplement: Supplementary Figure 1 — Box plots for miRNA and gene expression datasets after normalization. [file Data_Sheet_1.zip › Supplementary Figure 1.tif]

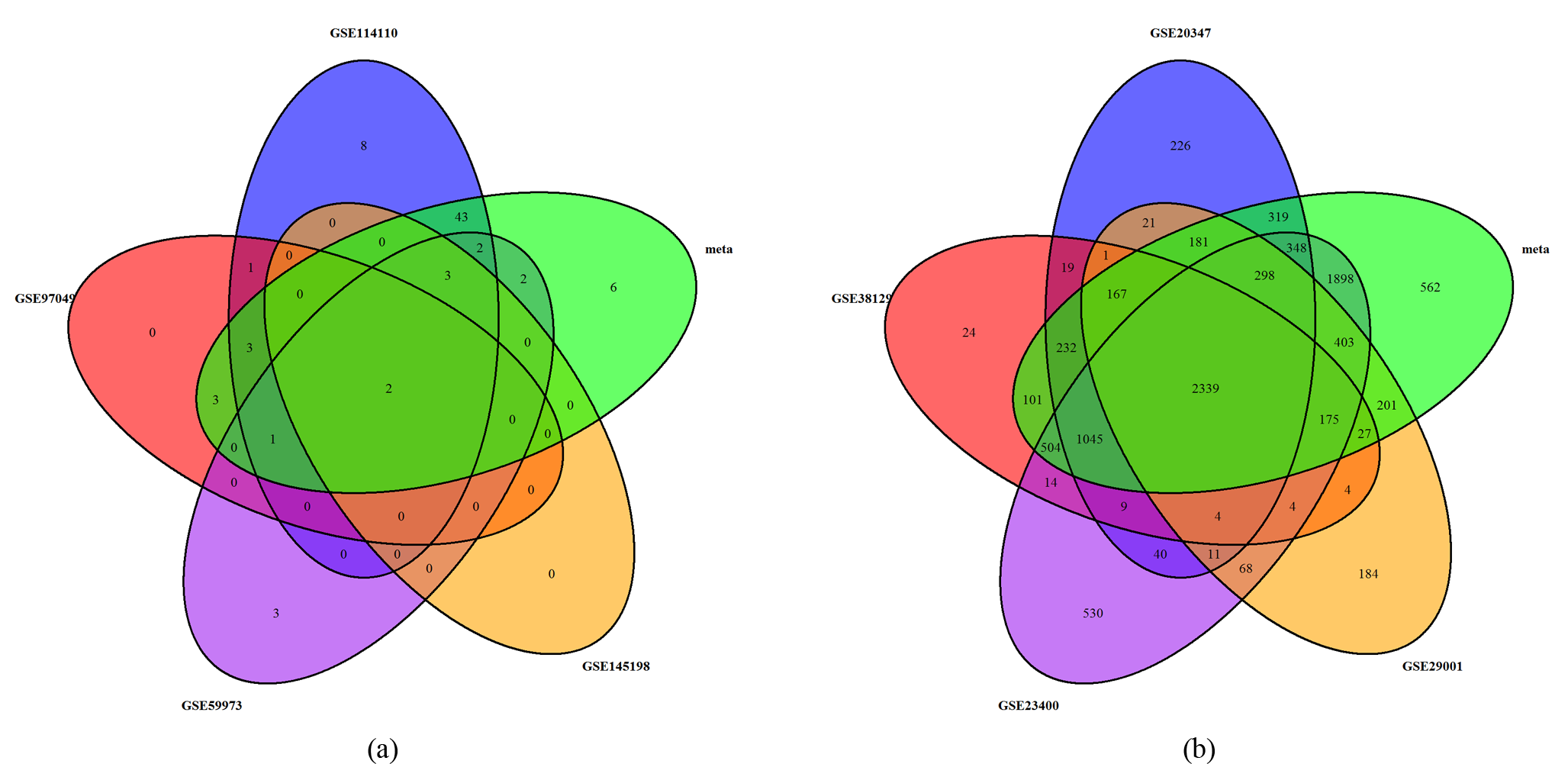

Supplement: Supplementary Figure 1 — Box plots for miRNA and gene expression datasets after normalization. [file Data_Sheet_1.zip › Supplementary Figure 2.tif]
